# Supplementary material for: Viral Loads in Ocular Fluids of Acute Retinal Necrosis Eyes Infected by Varicella-Zoster Virus Treated with Intravenous Acyclovir Treatment
Source: J Clin Med. 2020 Apr 22;9(4):1204. doi: 10.3390/jcm9041204 (PMC7230916; doi:10.3390/jcm9041204)
Supplement: Supplementary file 1 [file jcm-09-01204-s001.pdf]

Table 1.

Clinical summary of patients with suspected ARN and viral loads of ocular fluids

Pt. No.	Age	Gender	Laterality	Final diagnosis	Before antivirus treatment			Drug	Treatment period	During surgery					
					Sample	Pathogen	Viral load			Sample	Pathogen	Viral load	Sample	Pathogen	Viral load
1	40	F	L	**VZV**	Aqueous humor	VZV	1.73×10^{6}	**Aciclovir i.v.**	**4 days**						
2	45	M	R	**VZV**	Aqueous humor	VZV	8.87×10^{6}	**Aciclovir i.v.**	**6 days**						
3	75	M	R	**VZV**	Aqueous humor	VZV	3.00×10^{7}	**Aciclovir i.v.**	**8 days**						
4	50	M	R	**VZV**	Aqueous humor	VZV	6.00×10^{7}	**Aciclovir i.v.**	**3 days**						
5	64	M	L	**VZV**	Aqueous humor	VZV	5.23×10^{6}	**Aciclovir i.v.**	**7 days**				Vitreous fluid	VZV	4.30×10^{7}
6	47	F	L	**VZV**	Aqueous humor	VZV	1.23×10^{7}	**Valaciclovir oral**	**1 day**				Vitreous fluid	VZV	1.64×10^{8}
7	81	M	L	**VZV**	Aqueous humor	VZV	1.43×10^{8}	**Aciclovir i.v.**	**1 day**				Vitreous fluid	VZV	3.59×10^{8}
8	53	M	R	**VZV**	Aqueous humor	VZV	1.53×10^{8}	**Valaciclovir oral**	**5 days**				Vitreous fluid	VZV	1.03×10^{9}
9	67	M	L	**CMV**	Aqueous humor	CMV	1.74×10^{7}	**Aciclovir i.v.**	**1 day**				Vitreous fluid	CMV	6.10×10^{7}
10	64	M	L	**VZV**	Aqueous humor	VZV	2.47×10^{4}	**Aciclovir i.v.**	**3 days**				Vitreous fluid	VZV	1.26×10^{5}
11	45	F	L	**VZV**	Aqueous humor	VZV	4.62×10^{4}	**Aciclovir i.v.**	**8 days**				Vitreous fluid	VZV	2.05×10^{7}
12	86	F	L	**VZV**				**Aciclovir i.v.**	**1 day**				Vitreous fluid	VZV	1.06×10^{6}
13*	75	F	R	**VZV**				**Aciclovir i.v.**	**1 day**				Vitreous fluid	VZV	3.50×10^{7}
14*	75	F	L	**VZV**				**Aciclovir i.v.**	**5 days**				Vitreous fluid	VZV	4.40×10^{7}
15	83	F	L	**VZV**				**Vancomycin / Ceftazidime i.v.**	**0 day**				Vitreous fluid	VZV	2.41×10^{8}
16	41	M	L	**VZV**				**Aciclovir i.v.**	**2 day**	Aqueous humor	VZV	1.89×10^{7}	Vitreous fluid	VZV	2.40×10^{9}
17	77	F	R	**VZV**				**Aciclovir i.v.**	**2 day**	Aqueous humor	VZV	1.80×10^{7}	Vitreous fluid	VZV	7.80×10^{8}
18	50	M	R	**VZV**				**Aciclovir i.v.**	**2 day**	Aqueous humor	VZV	5.02×10^{7}	Vitreous fluid	VZV	7.47×10^{8}

Eighteen eyes of 17 patients with suspected ARN were enrolled. Eyes of No. 13 and No 14 belonged to same patient. In 4 cases (22.2%) of suspected ARN (upper rows with gray background), the pathogenic virus (VZV) was promptly confirmed by PCR test using aqueous humor samples collected before systemic antiviral treatments, and optimal antiviral treatments were initiated before therapeutic PPV. Viral load is given in units of copies/ml. PCR test was regarded positive in specimens with more than 5.0×10^{3} copies/ml, and the detected virus was confirmed as pathogen. ARN: acute retinal necrosis, CMV: cytomegalovirus, i.v.: intravenous drip, PCR: polymerase chain reaction, PPV: pars plana vitrectomy, VZV: varicella zoster virus, *: Eyes of same patient.

Table 2.

Sequences of primers and probes for detecting human herpes viruses using qualitative multiplex PCR

Herpes virus	Primer Sequence	Probe Sequence	Amplication	References
HSV-1 and HSV-2*	F: GCTCGAGTGCGAAAAAACGTTC<br>R: TGCGGTTGATAAACGCGCAGT	3'FITC: GCGCACCAGATCCACGCCCTTGATGAGC<br>LcRed604-5': CTTGCCCCCGCAGATGACGCC	polymerase	[19]
varicella zoster virus	F: TGTCCTAGAGGAGGTTTTATCTG<br>R: CATCGTCTGTAAAGACTTAACCAG	3'FITC: GGGAAATCGAGAAACCACCCTATCCGAC<br>LcRed640-5': AAGTTCGCGGTATAATTGTCAGT	gene 29	[20]
Epstein–Barr virus	F: CGCATAATGGCGGACCTAG<br>R: CAAACAAGCCCACTCCCC	3'FITC: AAAGATAGCAGCAGCGCAGC<br>LcRed640-5': AACCATAGACCCGCTTCCTG	BamH1	[21]
cytomegalovirus	F: TACCCCTATCGCGTGTGTTC<br>R: ATAGGAGGCGCCACGTATTC	3'FITC: TCGTCGTAGCTACGCTTACAT<br>LcRed705-5': ACACCACTTATCTGCTGGGCAGC	CMV glycoprotein	[22]
HHV type 6	F: ACCCGAGAGATGATTTTGCG<br>R: GCAGAAGACAGCAGCGAGAT	3'FITC: TAAGTAACCGTTTCGTCCCA<br>LcRed705-5': GGGTCATTTATGTTATAGA	101K gene region	[23]
HHV type 7	F: GAAAAATCCGCCATAATAGC<br>R: ATGGAACACCTATTAACGGC	3'FITC: GCCATAAGAAACAGGTACAGACATTGTCA<br>LcRed705-5': TTGTGAAATGTGTTGCG	U57	[24]
HHV type 8	F: AGCCGAAAGGATTCCACCAT<br>R: TCCGTGTTGTCTACGTCCAG	3'FITC: CCGGATGATGTAAATATGGCGGAAC<br>LcRed705-5': TGATCTATATACCACCAATGTGTCATTTATG	EB BDLF1 ORF26	[25]

The qualitative multiplex PCR for HSV detects both HSV type 1 and type 2 DNA in the same reaction. The optimized polymerase primer pair is common for HSV type 1 and type 2. On the other hand, the polymerase probe pair completely matches the genomic sequence of HSV type 2, but there are two base mismatches between the sequences of the probe pair and HSV type 1 genome. Therefore, the Tm of HSV type 1 is 15°C higher than that of HSV type 2, and the difference in Tm provides fine distinction between HSV type 1 and type 2. F: forward primer, HHV: human herpesvirus, HSV: herpes simplex virus, R: reverse primer

Table 3.

Sequences of primers and probes in human herpes viruses using quantitative real-time PCR

Herpes virus	Primer Sequence	Probe Sequence	Amplication	References
HSV type1	F: CGCATCAAGACCACCTCCTC<br>R: GCTCGCACCACGCGA	JOE-TGGCAACGCGGCCCAAC-TAMRA	gB	**[26]**
HSV type2	F: CGCATCAAGACCACCTCCTC<br>R: GCTCGCACCACGCGA	FAM-CGGCGATGCGCCCCAG-TAMRA	gB	
varicella zoster virus	F: AACTTTTACATCCAGCCTGGCG<br>R: GAAAACCCAAACCGTTCTCGAG	FAM-TGTCTTTCACGGAGGCAAACACGT-TAMRA	ORF29	**[27]**
Epstein–Barr virus	F: CGGAAGCCCTCTGGACTTC<br>R: CCCTGTTTATCCGATGGAATG	FAM-TGTACACGCACGAGAAATGCGCC-TAMRA	BALF5	**[28]**
cytomegalovirus	F: CATGAAGGTCTTTGCCCAGTAC<br>R: GGCCAAAGTGTAGGCTACAATAG	FAM-TGGCCCGTAGGTCATCCACACTAGG-TAMRA	IE-1	**[29]**
HHV type 6	F: GACAATCACATGCCTGGATAATG<br>R: TGTAAGCGTGTGGTAATGTACTAA	FAM-AGCAGCTGGCGAAAAGTGCTGTGC-TAMRA	U65-U66	**[30]**
HHV type 7	F: CGGAAGTCACTGGAGTAATGACAA<br>R: CCAATCCTTCCGAAACCGAT	FAM-CTCGCAGATTGCTTGTTGGCCATG-TAMRA	U37	**[31]**
HHV type 8	F: CCTCTGGTCCCCATTCATTG<br>R: CGTTTCCGTCGTGGATGAG	FAM-CCGGCGTCAGACATTCTCACAACC-TAMRA	ORF65	**[32]**

The quantitative real-time PCR for HSV is a multiplexing PCR that detects both HSV type 1 and type 2 DNA in the same reaction. The optimized gB primer pairs amplify both HSV type 1 and type 2 with equal efficiency, but the two type-specific probes are labeled with different fluorescent dyes. HSV type 1 probe is labelled with JOE at the 5′-end and with TAMRA at the 3′-end. HSV type 2 probe is labelled with FAM at the 5′-end and with TAMRA at the 3′-end.

Table 4.

Clinical characteristics of ARN patients with VZV infection classified into 4 groups according to the type of ocular fluid sample

Specimen	Pre-AH	Pre-AH and VF	VF	Post-AH and VF	*P* value
N	4	4	3	3	Among 4 goups
Age (year)	52.5 ± 15.5 (47.5)	63.5 ± 14.7 (64.0)	80.5 ± 7.78 (80.5)	56.0 ± 18.7 (50.0)	0.277
Gender (M / F)	3 / 1	3 / 1	0 / 2	2 / 1	0.531
Laterality (R / L)	3 / 1	0 / 4	1 / 2	2 / 1	0.531
Detection *N* (%)	4 (100)	4 (100)	3 (100)	3 (100)	–

ARN patients with VZV infection were divided into 4 groups according to the type of ocular fluid sample. There was no overlap of patients among 4 groups. Data are expressed as means ± standard deviations (median). Post-AH: aqueous humor samples collected during PPV after intravenous acyclovir treatment, Pre-AH: aqueous humor samples collected before the treatment, VF: vitreous fluid samples collected during PPV after the treatment. F: female, L: left, M: male, *N*: number, R: right.

Figure 1.

Representative results of a VZV-positive vitreous fluid sample (No. 11) analyzed using the combination PCR system

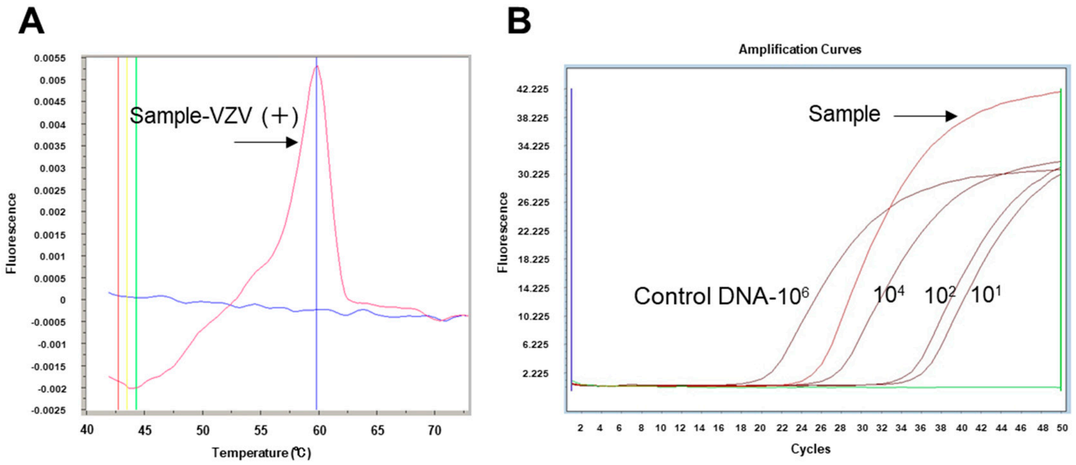

(A) Representative result of VZV-positive VF sample by qualitative multiplex PCR. After DNA extraction from the VF sample, multiplex PCR was performed to screen for viruses using LightCycler capillaries. At 60°C, a significant positive curve was detected, indicating the detection of VZV-DNA in the sample. At the same time, other HHVs such as HSV type 1, type 2 and CMV were confirmed to be negative for this sample. (B) Representative result of the same VZV-positive VF sample analyzed by quantitative real-time PCR (qPCR). The VZV genome copy number in the sample was calculated. The VF sample and control DNA (1.0×10^6, 10^4, 10^2 and 10^1 copies/ml) by the qPCR were tested, and a standard curve using the results of control DNA was generated. Values were considered to be significant when more than 10^3 copies/ml in the

sample were observed. In the qPCR assay, we conducted with positive controls, negative control (water), and sample in duplicate well assay. Representative single curves of each positive controls and sample are present in the graph. VF: vitreous fluid.

Figure 2.

Comparisons of viral loads in ocular fluids before and after initiation of intravenous acyclovir treatment

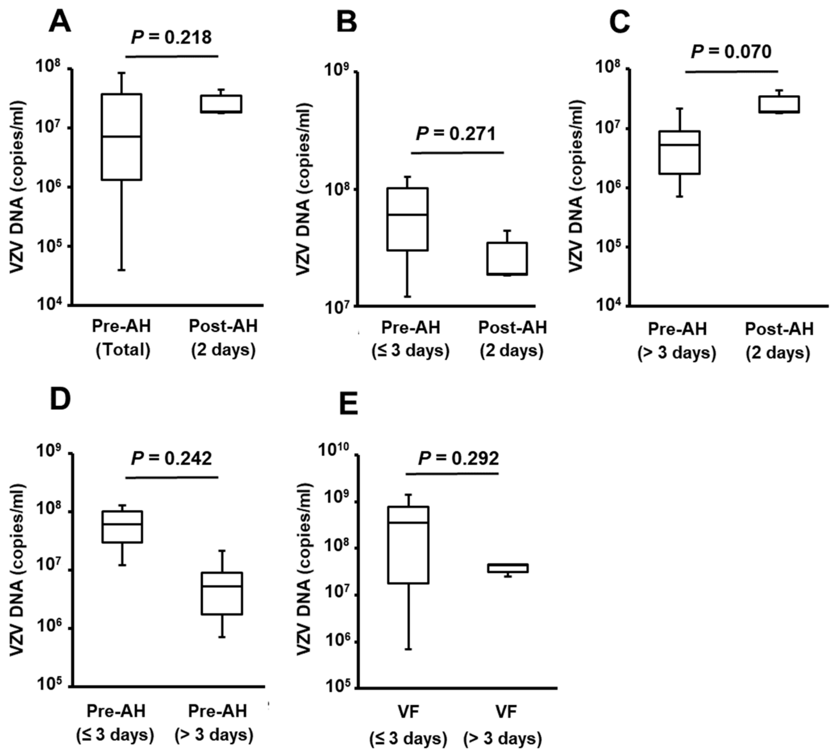

The box plot represents the median, 25/75 percentiles, and 10/90 percentiles. Total: all of Pre-AH samples collected before PPV, 2 days: sample collected 2 days after initiation of the treatment, ≤ 3 days: sample collected within 3 days after initiation of the treatment, > 3 days: sample collected more than 3 days after initiation of the treatment. Number of samples in each groups: Pre-AH (Total); 8 eyes, Pre-AH (≤ 3 days); 3 eyes, Pre-AH (> 3 days); 5 eyes, VF (≤ 3 days); 7 eyes, VF (> 3 days); 3 eyes.

Figure 3.

Comparison and correlation of viral loads in ocular fluids of the same patients before and after intravenous acyclovir treatment

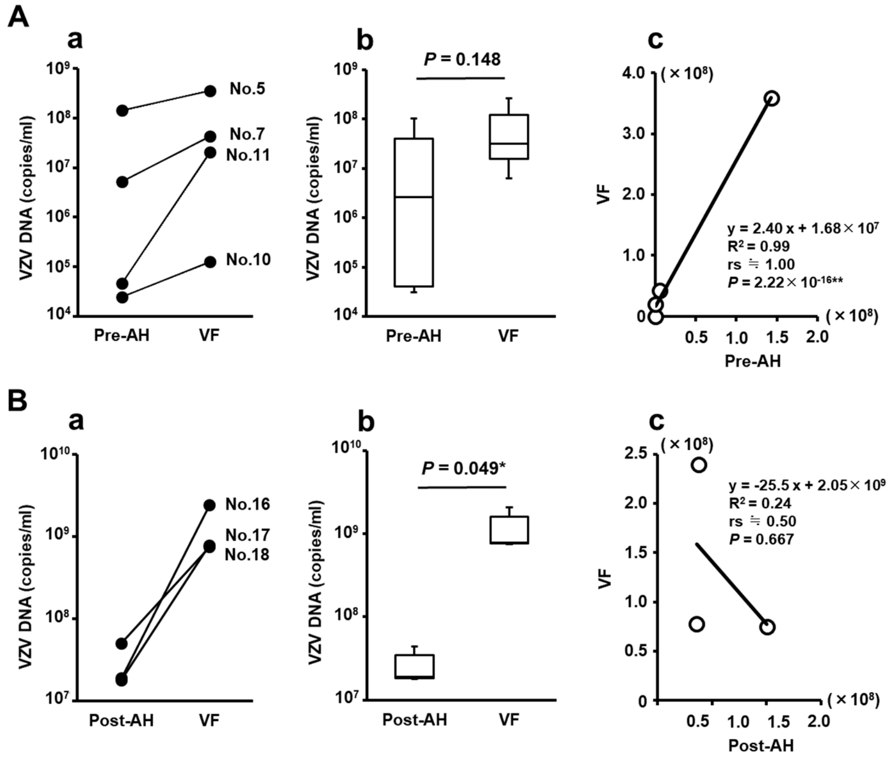

R^2: coefficient of determination, rs: Spearman's rank correlation coefficient, ≒: approximately equal, *: $P < 0.05$.

Supplemental Table 1.

Clinical characteristics of ARN patients with VZV infection classified into 5 groups according to type of ocular fluid sample and duration of intravenous acyclovir treatment

A

Sample	Aqueous humor		*P* value
Collection time	Pre (Total)	Post	
N	8	3	
Age (year)	58.0 ± 15.2 (57)	56.0 ± 18.7 (50)	0.461
Gender (M / F)	6 / 2	2 / 1	1.000
Laterality (R / L)	3 / 5	2 / 1	0.545
**Viral load (median)**	7.05×10^{6}	1.89×10^{7}	0.218
**Treatment period (days)**	5.00 ± 2.62 (5)	2	

B

Sample	Aqueous humor		*P* value
Collection time	Pre (≤ 3 days)	Post	
N	3	3	
Age (year)	65.0 ± 15.5 (64)	56.0 ± 18.7 (50)	0.209
Gender (M / F)	3 / 0	2 / 1	1.000
Laterality (R / L)	1 / 2	2 / 1	1.000
**Viral load (median)**	6.00×10^{7}	1.89×10^{7}	0.271
**Treatment period (days)**	2.33 ± 1.15 (3)	2	

C

Sample	Aqueous humor		*P* value
Collection time	Pre (> 3 days)	Post	
N	5	3	
Age (year)	53.8 ± 15.0 (45)	56.0 ± 18.7 (50)	0.335
Gender (M / F)	3 / 2	2 / 1	1.000
Laterality (R / L)	2 / 3	2 / 1	1.000
**Viral load (median)**	5.23×10^{6}	1.89×10^{7}	0.070
**Treatment period (days)**	6.60 ± 1.67 (7)	2	

D

Sample	Aqueous humor		*P* value
Collection time	Pre (≤ 3 days)	Pre (> 3 days)	
N	5	3	
Age (year)	65.0 ± 15.5 (64)	53.8 ± 15.0 (45)	0.140
Gender (M / F)	3 / 0	3 / 2	0.464
Laterality (R / L)	1 / 2	2 / 3	1.000
**Viral load (median)**	6.00×10^{7}	5.23×10^{6}	0.242
**Treatment period (days)**	2.33 ± 1.15 (3)	6.60 ± 1.67 (7)	

E

Sample	Vitreous fluid		*P* value
Collection time	Post (≤ 3 days)	Post (> 3 days)	
N	7	3	
Age (year)	67.7 ± 16.8 (75)	61.3 ± 15.2 (64)	0.224
Gender (M / F)	4 / 3	1 / 2	1.000
Laterality (R / L)	3 / 4	0 / 3	0.475
**Viral load (median)**	3.59×10^{8}	4.30×10^{7}	0.292
**Treatment period (days)**	1.71 ± 0.76 (2)	6.67 ± 1.53 (7)	

ARN patients with VZV infection were classified into 5 groups according to the type of ocular fluid sample and duration of durations of intravenous acyclovir treatment. Data are expressed as means ± standard deviations (median). Viral load is given in units of copies/ml. The data for comparisons A to E shown in this table are presented as box plots A to E, respectively in Figure 2. ARN: acute retinal necrosis, F: female, L: left, M: male, N: number, Post: samples collected during PPV after intravenous acyclovir treatment, PPV: pars plana vitrectomy, Pre: samples collected before the treatment, R:

right, VZV: varicella zoster virus, 2 days: samples collected 2 days after the treatment, ≤ 3 days: sample collected within 3 days after the treatment, > 3 days: sample collected more than 3 days after the treatment.

Supplemental Table 2.

Clinical characteristics of ARN patients with VZV infection divided into 2 groups according to type of paired sample in ocular fluid

A

**Sample**	**Pre-AH**	**VF**	***P* value**
N	**4**		
Age (year)	63.5 ± 14.7 (64)		
Gender (M / F)	3 / 1		
Laterality (R / L)	0 / 4		
**Viral load (median)**	**2.64×10^6**	**3.18×10^7**	**0.148**
**Treatment period (days)**	**4.75 ± 3.30 (5)**		

B

**Sample**	**Post-AH**	**VF**	***P* value**
N	**3**		
Age (year)	56.0 ± 18.7 (50)		
Gender (M / F)	2 / 1		
Laterality (R / L)	2 / 1		
**Viral load (median)**	**1.89×10^7**	**7.90×10^8**	**0.049***
**Treatment period (days)**	**2**		

ARN patients with VZV infection were divided into 2 groups according to the type of paired sample in ocular fluid. The data for comparisons of A and B shown in this table are presented as box plots A-[b] and B-[b], respectively in Figure 3. *: $P < 0.05$.
